# Evaluation of dimethyl sulfoxide (DMSO) as a mobile phase additive during top 3 label-free quantitative proteomics

**DOI:** 10.1016/j.ijms.2015.07.004

**Published:** 2015-11-30

**Authors:** Dominika Strzelecka, Stephen W. Holman, Claire E. Eyers

**Affiliations:** a Division of Biophysics, Institute of Experimental Physics, Faculty of Physics, University of Warsaw, Zwirki i Wigury 93, 02-089 Warsaw, Poland; b Centre for Proteome Research, Department of Biochemistry, Institute of Integrative Biology, University of Liverpool, Crown Street, Liverpool L69 7ZB, UK

**Keywords:** cpc, copies per cell, DIA, data-independent acquisition, DMSO, dimethyl sulfoxide, ESI, electrospray ionisation, HDMS^E^, ion mobility-assisted MS^E^, LC, liquid chromatography, MS, mass spectrometry, MS/MS, tandem mass spectrometry, MS^E^, mass spectrometry with elevated energy (a form of data-independent tandem mass spectrometry), Q-ToF, quadrupole-time-of-flight, SWATH, sequential window acquisition of all theoretical fragment ion spectra, Proteomics, Quantification, DMSO, Label-free, Peptide, LC–MS

## Abstract

Dimethyl sulfoxide (DMSO) has been advocated as a beneficial additive to electrospray solvents for peptide analysis due to the improved ionisation efficiency conferred. Previous reports have shown that the resultant improvements in peptide ion signal intensities are non-uniform. As a result, it was hypothesised that inclusion of DMSO in electrospray solvents could be detrimental to the outcome of intensity-based label-free absolute quantification approaches, specifically the top 3 method. The effect of DMSO as a mobile phase additive in top 3 label-free quantification was therefore evaluated. We show that inclusion of DMSO enhances data quality, improving the precision and number of proteins quantified, with no significant change to the quantification values observed in its absence.

## Highlights

• Top 3 label-free quantification is compatible with DMSO in the LC mobile phases.
• Significantly different populations of peptides identified in the presence of DMSO.
• The precision of protein quantification and the number of proteins quantified improve with DMSO.

## Graphical abstract

**Figure:**
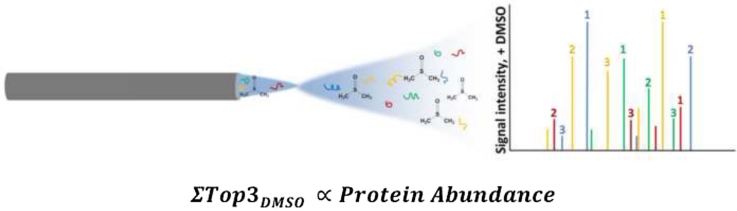

Liquid chromatography–tandem mass spectrometry (LC–MS/MS) is the analytical technique of choice for large scale bottom-up proteomics analyses [1]. Peptide-based quantification is now becoming a routine requirement for many laboratories [2], and for reasons of cost-effectiveness, simpler sample preparation, capability for highly multiplexed experiments and the potential for extensive proteome coverage with quantitative information, label-free quantification strategies have become popular in the field [3]. One widely applied label-free quantification approach is the top 3 method (typically exploited as part of a data-independent acquisition (DIA) workflow), first reported nearly a decade ago by Silva and co-workers [4]. In this approach, the summed intensity of the three best ionising peptides (ΣTop3) *i.e.* those with the highest signal intensities, for a protein is used as a proxy for its quantity. By determining this value for a known quantity of calibrant protein added to the sample(s) under investigation, an instrumental response factor can be established. Using this factor, absolute protein levels can subsequently be determined for the constituents of the complex sample based on the individual protein ΣTop3. This method has been shown to be suitable for quantifying proteins over four orders of magnitude and measuring fold changes in a sensitive manner [5].

Recently, it has been reported that the addition of low percentages of dimethyl sulfoxide (DMSO) (≤5%) to the solvents during nano-electrospray (nESI) ionisation of peptides results in improved ionisation efficiency and coalescence of ion current into fewer charge states [6], [7]. Consequently, higher quality product ion spectra are acquired, which leads to greater numbers of peptide, and concomitantly protein, identifications. Furthermore, the increase in signal intensity means that lower limits of detection (and thus quantification) are possible, with improved accuracy and precision due to enhanced ion statistics. However, previous work has shown that the improvement in peptide signal intensities is non-uniform [7]. Therefore, it was hypothesised that the top 3 approach for absolute protein quantification may no longer be applicable upon inclusion of DMSO, given that the method relies on the signal intensity measurements of three separate peptides for each protein. To test this hypothesis, yeast cells were grown in biological quadruplicate, lysed and digested using trypsin as previously described [8]. Samples were analysed in triplicate by LC–MS^E^ using a Waters nanoACQUITY nano-uHPLC instrument coupled to a Waters Synapt HDMS instrument (see supplementary material for full details) without and with DMSO (added to 3%, previously determined to be optimal for Waters instruments [7]) present in the LC mobile phases. Fig. 1 shows the overlap of peptide identifications and protein quantifications in the absence and presence of DMSO. A peptide or protein was deemed identified/quantified respectively if it was recorded in at least two of three technical replicates for at least three of four biological replicates. Increases in identifications at both the peptide- and protein-level (3.3% and 14.9% respectively) were observed, which were broadly similar in magnitude to that observed in other recent studies [9], [10]. The increases were lower than those reported in the original work of Hahne et al. [7], predominantly due to differences in their approach to data handling compared to the current work, *i.e.* summing nonredundant identifications from technical triplicate analyses *cf.* removing peptides only observed once out of three technical replicates. When our data were manipulated in a manner analogous to that of Hahne and co-workers, the increases in identifications in the presence of DMSO rose to 20.2% and 16.2% (peptides and proteins respectively, averaged over four biological replicates). This compares much more favourably with the data originally reported, with the number of peptides and proteins observed increasing by 35% and 28% respectively but on a much longer LC gradient (210 min *versus* 90 min). Moreover, lower gains would be expected here given the previous finding that the DMSO-mediated increases in signal intensity on Thermo instruments (as used in the study by Hahne et al.) were greater than on Waters instruments (as was employed here) [7]. Evaluation of the signal intensities of the 1331 peptides identified both in the absence and presence of DMSO showed that, as expected, the detectability of the vast majority improved with DMSO (on average over 27%), evinced by their position above the *y* = *x* line (Fig. 2, Supplementary Information Figure S1). Consistent with the previous reports the change in signal intensity is non-uniform over the population of peptides [6], [7], confirmed by the *R*^2^ value of 0.836 (Fig. 2); peptide ions of lower signal intensity exhibit greater benefit (higher relative increase in signal intensity) upon addition of DMSO.

Interestingly, different populations of peptides (and proteins) were identified under the two conditions (Fig. 1). Assessment of the total number of acidic residues (aspartic acid and glutamic acid) showed a significant enrichment for more acidic peptides when DMSO was present in the mobile phases (*p* = 1 × 10^−3^ using the Mann–Whitney *U* test). The enhanced identification of more acidic peptides has previously been attributed to the reduction in competition for ionisation as result of sequestration of single analyte molecules into charged droplets during the ESI process [7]. Furthermore, peptides with higher numbers of hydrophobic residues (alanine, isoleucine, leucine, phenylalanine, tryptophan and valine) were also significantly enriched in the presence of DMSO (*p* = 2 × 10^−4^ using the Mann–Whitney *U* test). This is likely due to the additional organic solvent in the aqueous mobile phase compared to the experimental set-up without DMSO, leading to enhanced elution of more lipophilic peptides. Combined, these observations demonstrate that the addition of DMSO to LC mobile phases allows a different region of peptide chemical space to be interrogated and thus can provide a means of complementary analysis to that of ‘standard’ LC–MS.

The three highest peptide intensity measurements for each protein (ΣTop3) were used to determine the quantity (in femtomoles) of the parent protein in the sample with reference to an exogenous standard, rabbit glycogen phosphorylase B. This quantity was converted into copies per cell (cpc) to normalise the data across the four biological replicates. Fig. 3 shows the comparison of the cpc values for 233 of the 238 proteins that were identified both with and without DMSO in the LC mobile phases. The five most abundant proteins were removed from the data set due to detector saturation effects (as can be observed on mass spectrometers with time-to-digital converters (TDC), such as that used in this study) [11], which affected the linearity of response and thus the computed cpc value (Supplementary material Figure S2). Over the two orders of magnitude within which the cpc values correlate linearly (*R*^2^ = 0.949), the gradient of the line was almost exactly unity and no statistically significant difference between the two data sets was observed (*p* = 0.054 using the Mann–Whitney *U* test). The absolute quantitative measurements in the presence of DMSO are thus globally unchanged compared to those obtained in its absence for this data set, indicating that the top 3 quantification method is compatible with inclusion of DMSO in the LC mobile phases. The non-uniform increase in peptide intensities is averaged out over the three peptides used for absolute protein quantification, resulting in the same protein-level quantitative value. The addition of DMSO to the LC mobile phases also lead to a statistically significant increase in precision, with the median relative standard deviation, in terms of cpc, decreasing from 21% to 15% (*p* = 2 × 10^−6^ using the Mann–Whitney *U* test).

## Conclusions

The data presented show that the addition of DMSO to LC mobile phases does not detrimentally affect the outcome of a top 3 label-free absolute protein quantification experiment. Absolute quantification values (cpc) for 233 yeast proteins in the absence and presence of DMSO showed a linear relationship of almost unity (*y* = 1.06*x*) with high correlation (*R*^2^ = 0.949) and no statistically significant difference. Indeed, data quality was improved when DMSO was employed in the experiment, with an increase in both peptide identifications (3.3%) and protein quantifications (14.9%) and a statistically significant improvement in the precision of the quantitative measurements. Addition of DMSO to LC mobile phases is thus recommended to improve the outcome of a top 3 label-free proteomics quantification experiment when performed on a Q-ToF mass spectrometer. Although it remains to be seen whether similar observations will be made using alternative instrumentation, preliminary investigations using a Thermo LTQ Orbitrap Velos suggest a similar trend for data-dependent analyses on the different platform (data not shown). It is anticipated therefore that the beneficial effects of DMSO for protein quantification will be realised using other, both data-dependent and data-independent, acquisition strategies (such as SWATH – sequential window acquisition of all theoretical fragment ion spectra) [12]. Additionally, given that the beneficial effects of DMSO take place at the point of ionisation, such methodology should also be applicable to MS^E^ assisted by ion-mobility separation (HDMS^E^) [13].

## Figures and Tables

**Fig. 1:**
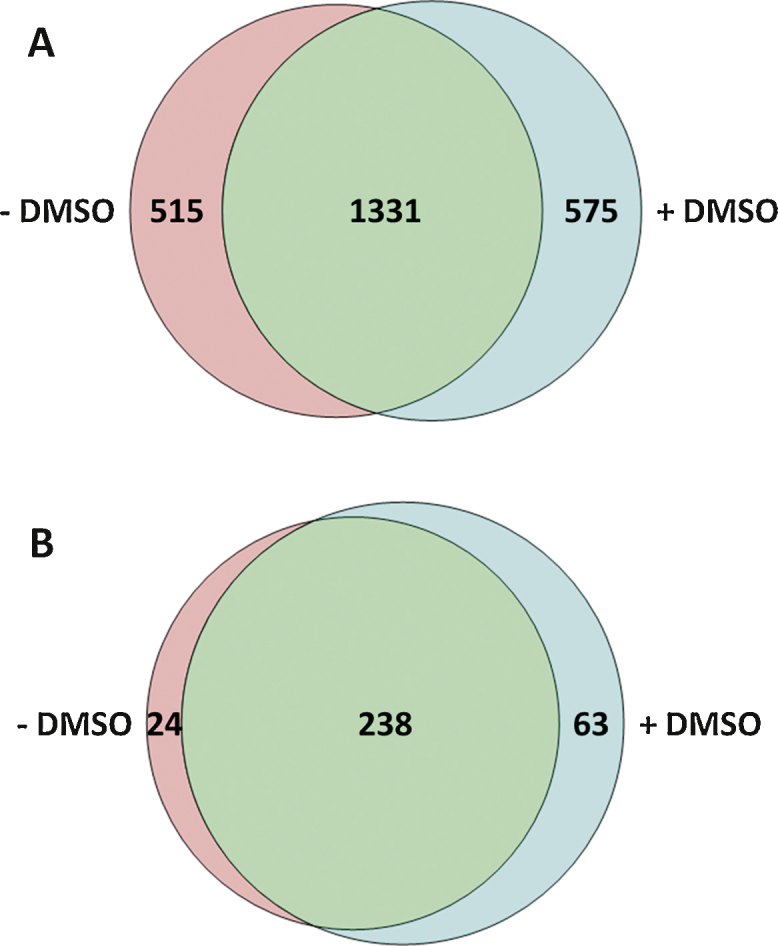
Venn diagrams displaying the number and overlap of (A) identified peptides, and (B) quantified proteins without and with 3% DMSO present in the LC mobile phases.

**Fig. 2:**
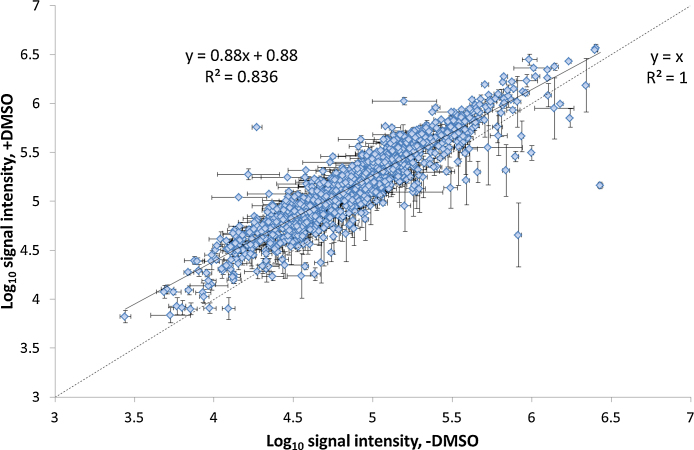
Log_10_ average peptide signal intensities in the absence and presence of DMSO in the LC mobile phases for the 1331 yeast peptides identified under both conditions. Error bars represent ±standard error of the log_10_ mean for all the replicate measurements under each condition (both biological and technical replication, *n* ≥ 6).

**Fig. 3:**
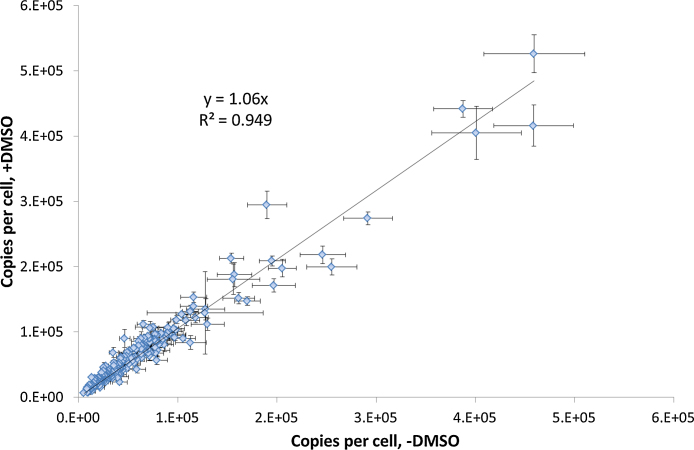
Average protein copies per cell in the absence and presence of DMSO in the LC mobile phases for 233 of the yeast proteins quantified under both conditions. Error bars represent ±standard error of the mean for the four biological replicate measurements made under each condition.
