# Supplementary material for: Evaluation of dimethyl sulfoxide (DMSO) as a mobile phase additive during top 3 label-free quantitative proteomics
Source: Int J Mass Spectrom. 2015 Nov 30;391:157–60. doi: 10.1016/j.ijms.2015.07.004 (PMC4708063; doi:10.1016/j.ijms.2015.07.004)
Supplement: Supplementary file 1 [file mmc1.docx]

Evaluation of dimethyl sulfoxide (DMSO) as a mobile phase additive during top 3 label-free quantitative proteomics

Supplementary material

Dominika Strzelecka^1,2,#^, Stephen W. Holman^1,#^ and Claire E. Eyers^1*^

^1^ Division of Biophysics, Institute of Experimental Physics, Faculty of Physics, University of Warsaw, Zwirki I Wigury 93, 02-089, Warsaw, Poland

^2^ Centre for Proteome Research, Department of Biochemistry, Institute of Integrative Biology, University of Liverpool, Crown Street, Liverpool, L69 7ZB, UK

# These authors contributed equally to the work

*Address for correspondence: Professor Claire E. Eyers

Institute of Integrative Biology

University of Liverpool Crown Street

Liverpool

L69 7ZB

UK

Email: [claire.eyers@liverpool.ac.uk](mailto:claire.eyers@liverpool.ac.uk)

Tel: +44 151 795 4424

**Experimental**

*Cell lysis and protein extraction*

Yeast (*S. cerevisiae*, EUROSCARF accession number Y11335 BY4742; *MAT*α; *his3Δ1*; *leu2Δ0*; *lys2Δ0*; *ura3Δo*; *YJL088w*::*Kan*MX4) was prepared as previously described.[1] In brief, cells were grown in chemostat mode (dilution rate of 0.1 h^-1^) carbon-limiting F1 medium using 10 g L^-1^ of glucose as the sole carbon source, with additional arginine (0.5 mM) and lysine (1 mM) to meet the auxotrophic requirements of the strain. Aliquots (15 mL) were harvested and the cell count determined using an AUTO M10 cellometer (Nexcelom Bioscience, Lawrence, MA, USA) prior to centrifugation (4000 rpm, 4 ^o^C, 10 min). The supernatant fraction was discarded, the pellet flash frozen in liquid nitrogen and stored at -80 ^o^C prior to protein extraction.

To extract the protein, cells were reconstituted in 250 µL of 50 mM ammonium bicarbonate (AmBic) containing a mini-protease inhibitor tablet with EDTA (Roche, Welwyn Garden City, UK) and transferred to 2 mL screw cap vials. Acid-washed beads (250 µL) were added and the pellets subjected to repeated rounds of bead beating (15 x 30 s with 1 min cool down between each cycle) at 4 ^o^C. The broken cells were centrifuged (13,000 rpm, 4 ^o^C, 10 min) and the supernatant fraction collected in low-binding microcentrifuge tubes on ice. A second 250 µL aliquot of 50 mM AmBic/protease inhibitor buffer was added and the pellet reconstituted by vortexing. The bottom of the screw cap vial was pierced with a hot needle, the vial placed inside a clean tube and then centrifuged (4000 rpm, 4 ^o^C, 10 min). The flow-through was combined with the supernatant fraction, mixed by vortexing and the final volume recorded. The protein concentration was determined using a Bradford assay (Bio-Rad, Hemel Hempstead, UK). The broken cell preparations were stored at -80 ^o^C prior to protein digestion.

*Protein digestion*

Whole cell yeast lysates in biological quadruplicate were subjected to proteolysis using trypsin. One hundred micrograms of each replicate were solubilised in 160 µL of 25 mM ammonium bicarbonate. Proteins were denatured by the addition of *Rapi*Gest SF (Waters Ltd., Elstree, UK) to 0.05 % (10 µL of 1 % (w/v)) and heating at 80 ^o^C for 10 min. Disulfide bonds were reduced by dithiothreitol added to 3 mM (10 µL of 60 mM, 60 ^o^C, 10 min) and the formed thiol groups were alkylated through the addition of iodoacetamide to 9 mM (10 µL of 180 mM room temperature in the dark, 30 min). Proteomics grade trypsin was added to 2 % (w/w; 10 µL of 0.2 µgµL^-1^) and the samples incubated at 37 ^o^C for 4.5 h. A second addition of proteomics grade trypsin was added to 2 % (w/w; 10 µL of 0.2 µgµL^-1^) and the samples incubated overnight at 37 ^o^C. Trifluoroacetic acid (1 µL) was added (0.5 % (v/v) final concentration) and the samples incubated at 37 ^o^C for 2 h to precipitate the insoluble *Rapi*Gest SF hydrolysis product, which was cleared by centrifugation at 13,000 *g* for 15 min.

*LC-MS/MS analysis*

A portion of each yeast biological replicate was mixed with rabbit glycogen phosphorylase B (GPB) (Waters Ltd., Elstree, UK) to a ratio of 450 ng: 50 fmol in 0.1 % formic acid in H_2_O:MeCN [97:3]. Each sample was analysed in triplicate (450 ng yeast tryptic digest + 50 fmol GPB loaded on column for each analysis) under both sets of conditions *i.e.* with and without DMSO. The injection order of the samples was randomised.

LC-MS/MS analysis was performed using a nanoACQUITY ultra-performance liquid chromatography system (Waters Ltd., Elstree, UK) (operated in dual pump trapping mode to avoid DMSO being present in the trapping solvent and enable retention of hydrophilic peptides through the use of a predominantly aqueous solvent mixture) and a Synapt HDMS Q-TOF mass spectrometer (Waters Ltd., Elstree, UK). Both instruments were operated under the control of MassLynx v4.1. The samples were loaded onto a Symmetry C18 trapping column (5 µm packing material, 180 µm x 20 mm) (Waters Ltd., Elstree, UK) in 0.1 % FA, 0.1 % MeCN (5 µL min^-1^) for 3 min prior to gradient elution onto a HSS T3 nanoACQUITY C18 analytical column (1.8 µm packing material, 75 µm x 200 mm) (Waters Ltd., Elstree, UK). The gradient starting conditions were 97 % A (either 0.1 % FA or 0.1% FA, 3 % DMSO): 3 % B (either 0.1 % FA in MeCN or 0.1 % FA, 3 % DMSO in MeCN). The column was developed at 300 nL min^-1^ to 40 % B over 90 min, then to 95 % B over 2 min, held at 95 % B for 2.5 min and then re-equilibrated. The column oven temperature was 35 ^o^C and the autosampler temperature was 4 ^o^C. A lock mass solution of 500 fmol µL^-1^ of glu-fibrinopeptide B in 0.1 % FA in H_2_O:MeCN [50:50] was infused into the nano-ESI source from an auxiliary pump at a constant flow rate of 300 nL min^-1^. All solvents were LC-MS grade.

The column effluent was introduced into a nano-ESI source fitted with a PicoTip emitter (New Objective, Woburn, MA, USA). The polarity of the ionisation source was set to positive. The ionisation source was operated under the following conditions; capillary voltage, 3.0 kV; cone voltage, 25 V; extraction cone, 4 V; source temperature, 80 ^o^C (absence of DMSO) or 150 ^o^C (presence of DMSO); trap gas 1.5 mL min^-1^; detector 2000 V. The instrument was calibrated immediately prior to analysis of the samples using the product ion spectrum of glu-fibrinopeptide B (500 fmol µL^-1^ in 0.1 % FA in H_2_O:MeCN [50:50]), which was infused directly into the nano-ESI source at a constant flow rate of 500 nL min^-1^. Data acquisitions were performed using a MS^E^ experiment with the instrument in V mode (single pass reflectron), affording an approximate resolution of 10,000 FWHM. A survey scan was performed over the *m*/*z* range 50-2000 with a scan time of 1 sec and a trap cell collision energy of 6 eV. Product ion spectra were then acquired over the *m*/*z* range 50-2000 with a scan time of 1 sec. The trap cell collision energy was ramped from 15 to 40 eV over the course of the scan. The transfer cell collision was maintained at 4 eV for both the survey and product ion scans. The lock mass was sampled every 30 sec using a trap cell collision energy of 6 eV and a cone voltage that facilitated a detector response of between 100-200 counts-per-second.

*Data processing*

Data was processed using ProteinLynx Global Server (PLGS) v2.5.2 (Waters Ltd., Elstree, UK). The data preparation stage thresholds were: low energy, 250 counts; elevated energy, 100 counts; intensity threshold, 1500 counts. The lock mass was defined as glu-fibrinopeptide B ([M + 2H]^2+^, *m*/*z* 785.8426). The processed spectra were searched against the UniProt reference complete proteome set for *S. cerevisiae* (strain ATCC 204508/S288c, downloaded 14^th^ August 2014). The sequence of rabbit glycogen phosphorylase B (GPB) was added to the database and a reversed database created. Automatic settings for precursor and product ion mass tolerance were used, and thresholds for a match were as follows: minimum fragment ion matches per peptide, 3; minimum fragment ion matches per protein, 7; minimum peptide matches per protein, 1. A fixed carbamidomethyl modification for cysteine and variable modifications for methionine oxidation and *N*-terminal acetylation were specified. Two trypsin missed cleavages were allowed to account for the high number of potential missed cleavages sites and dibasic sequence motifs in the yeast proteome.[2] The threshold score/expectation value for the acceptance of individual spectra was the default value for the program, such that the false positive rate at the protein-level was 4 %. Quantification was performed by the software using the top3 methodology with GPB as the calibration protein.[3] The output from PLGS v.2.5.2 was filtered so that only peptides and proteins identified/quantified respectively in at least two out of three technical replicates and at least three out of four biological replicates were retained in the data set. The filtering of the technical replicate data led to a true protein-level false positive rate of ≤ 0.71 % for each individual biological replicate.

**Observations on the use of DMSO as a mobile phase additive**

Over the course of the data acquisition (~ one month), no deleterious effects to the hardware used were observed when using high purity DMSO (Sigma-Aldrich, catalogue number 34869). An increase in low molecular weight background (< 350 amu) was seen, but with no detriment to the data acquired in this work.

The use of DMSO necessitated a higher ionisation source temperature for optimal performance. An infusion of glu-fibrinopeptide B was performed to determine the optimal temperature for desolvation in both the absence and presence of DMSO. In the absence of DMSO, increasing the temperature above 80 ^o^C (the standard operating temperature for Synapt instrument used in this study) did not increase peptide ion signal intensity. However, inclusion of DMSO to the solvent mixture required a higher ionisation source temperature for efficient desolvation, with peptide ion current increasing with increasing temperature (as assessed by monitoring the signal intensity of the [M+2H]^2+^ ion of glu-fibrinopeptide B). The maximum permissible ionisation source temperature on the Synapt HDMS is 150 ^o^C, so this was used for all experiments when DMSO was present in the mobile phases. Optimisation and consistent ionisation source temperature ensured that a like-for-like comparison was conducted in respect of desolvation during ESI.


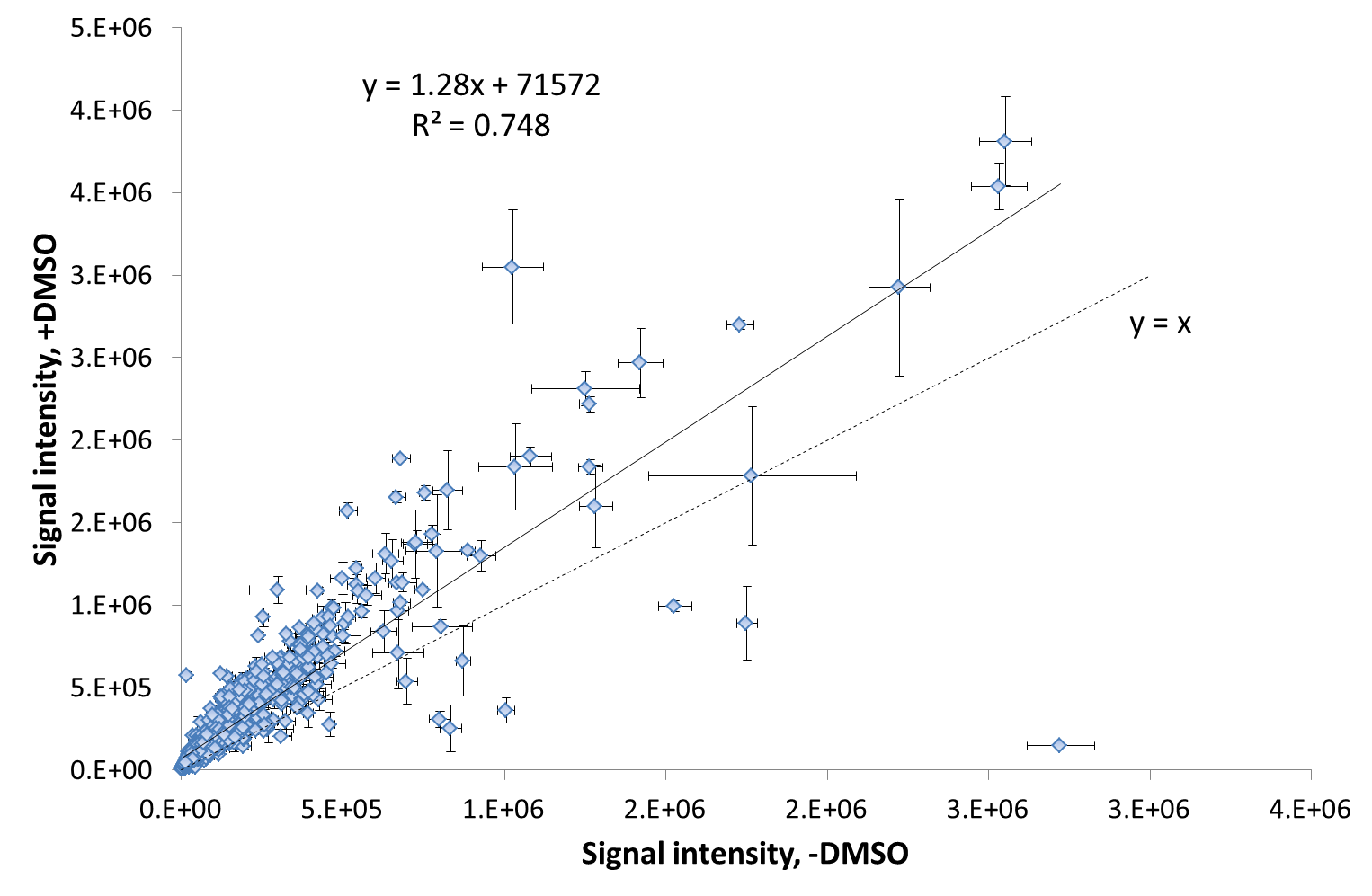


Figure S1 Average peptide signal intensities in the absence and presence of DMSO in the LC mobile phases for the 1331 yeast peptides identified under both conditions. Error bars represent ± standard error of the mean for the replicate measurements under each condition (both biological and technical replication, n ≥ 6)


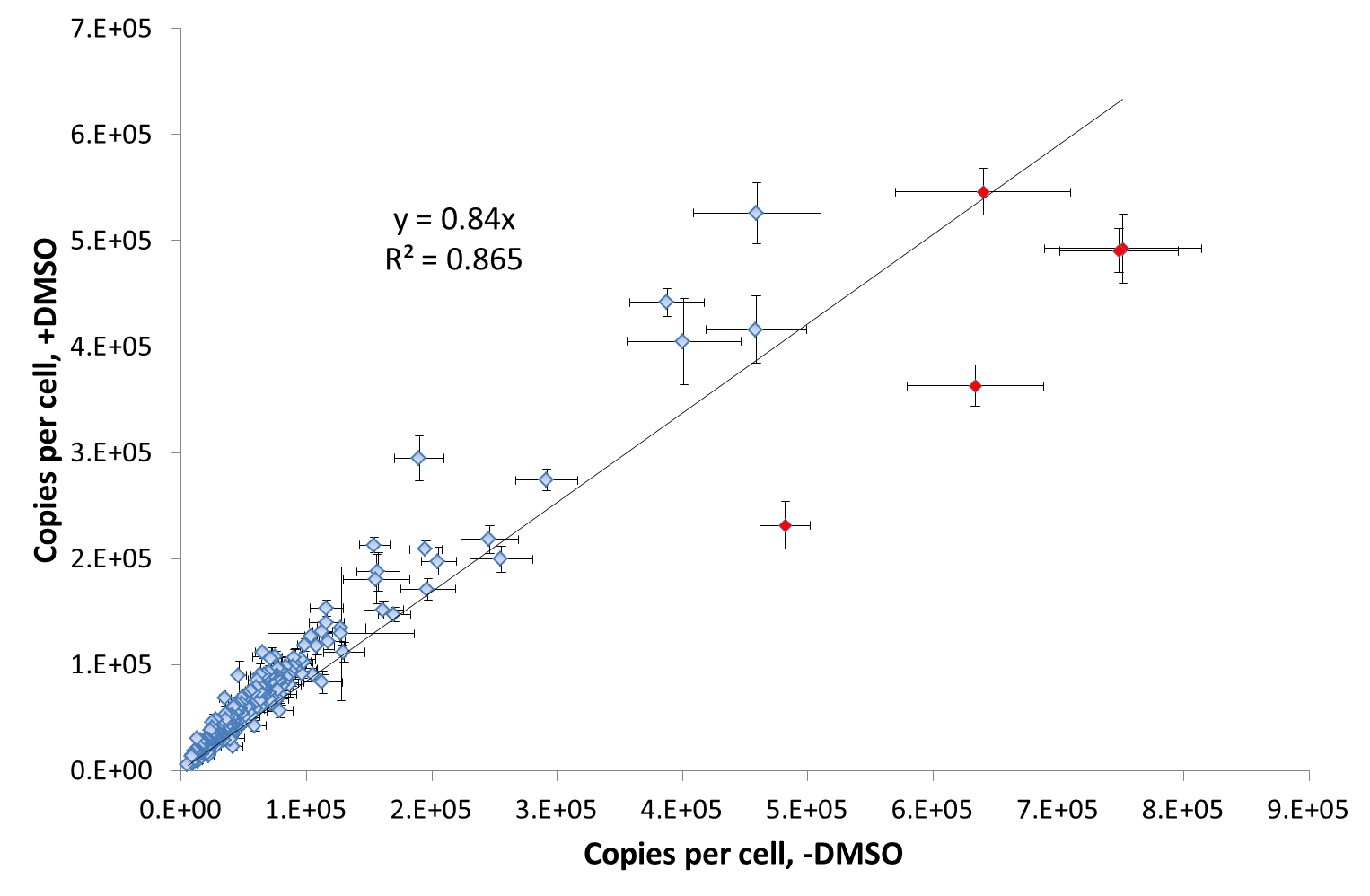


Figure S2 Average protein copies per cell in the absence and presence of DMSO in the LC mobile phases for 238 yeast proteins quantified under both conditions. Error bars represent ± standard error of the mean for the four biological replicate measurements made under each condition. The red data points represent those removed from the final data set due to detector saturation to generate Figure 3.

References

[1] P. Brownridge, C. Lawless, A.B. Payapilly, K. Lanthaler, S.W. Holman, V.M. Harman, C.M. Grant, R.J. Beynon, S.J. Hubbard, Quantitative analysis of chaperone network throughput in budding yeast, Proteomics, 13 (2013) 1276-1291.

[2] C. Lawless, S.J. Hubbard, Prediction of misssed proteolytic cleavages for the selection of surrogate peptide for quantitative proteomics, OMICS, 16 (2012) 449-456.

[3] J.C. Silva, M.V. Gorenstein, G.-Z. Li, J.P.C. Vissers, S.J. Geromanos, Absolute quantification of proteins by LC-MS^E^: A virtue of parallel MS acquisition, Mol. Cell. Proteomics, 5 (2006) 144-156.
